# Selective antibacterial effects of mixed ZnMgO nanoparticles

**DOI:** 10.1007/s11051-013-1595-4

**Published:** 2013-04-06

**Authors:** Jasmina Vidic, Slavica Stankic, Francia Haque, Danica Ciric, Ronan Le Goffic, Aurore Vidy, Jacques Jupille, Bernard Delmas

**Affiliations:** 1 VIM, Institut de la Recherche Agronomique, Jouy en Josas, France; 2 CNRS, Institut des Nanosciences de Paris, UMR 7588, 4 Place Jussieu, 75252 Paris Cedex 05, France; 3 UPMC, Université Paris 06, INSP, UMR 7588, 4 Place Jussieu, 75252 Paris Cedex 05, France; 4 Department of Ecology, Institute for Biological Research “Sinisa Stankovic”, University of Belgrade, 11000 Belgrade, Serbia

**Keywords:** Metal oxide nanoparticles, ZnMgO, Antibacterial activity, *E. coli*, *B. subtilis*, Nanoparticle toxicity

## Abstract

Antibiotic resistance has impelled the research for new agents that can inhibit bacterial growth without showing cytotoxic effects on humans and other species. We describe the synthesis and physicochemical characterization of nanostructured ZnMgO whose antibacterial activity was compared to its pure nano-ZnO and nano-MgO counterparts. Among the three oxides, ZnO nanocrystals—with the length of tetrapod legs about 100 nm and the diameter about 10 nm—were found to be the most effective antibacterial agents since both Gram-positive (*B. subtilis*) and Gram-negative (*E. coli*) bacteria were completely eradicated at concentration of 1 mg/mL. MgO nanocubes (the mean cube size ~50 nm) only partially inhibited bacterial growth, whereas ZnMgO nanoparticles (sizes corresponding to pure particles) revealed high specific antibacterial activity to Gram-positive bacteria at this concentration. Transmission electron microscopy analysis showed that *B. subtilis* cells were damaged after contact with nano-ZnMgO, causing cell contents to leak out. Our preliminary toxicological study pointed out that nano-ZnO is toxic when applied to human HeLa cells, while nano-MgO and the mixed oxide did not induce any cell damage. Overall, our results suggested that nanostructured ZnMgO, may reconcile efficient antibacterial efficiency while being a safe new therapeutic for bacterial infections.

## Introduction

Common pathogens, especially bacteria, remain a major health concern responsible for causing a large number of deaths and hospitalizations each year. The discovery of antibiotics in the 1940s saved the lives of millions of people. However, because of the widespread and sometimes inappropriate use of antibiotics, strains of bacteria have begun to gain resistance to this type of therapeutic. In industrialized countries, bacteria are developing multiple resistances to a range of antibiotics, which triggers a greater need for efficient antimicrobial agents to which bacteria might not develop resistance.

Over the past decade, many potential antibacterial agents—including nanometer-sized metal oxides—have been researched. Some of these agents were found to be cytotoxic against bacteria but not against mammalian cells making, thus, medical applications possible (Taylor and Webster 2011). The use of inorganic nanoparticles has attracted lots of interest mostly because of their reliable antimicrobial activity found to be effective at low concentrations (Anagnostakos et al. 2008). This is due to their high specific surface area which allows a broad range of reactions with the bacterial surface. Therefore, it is not surprising that the antibacterial activity of metal oxide nanoparticles is size-dependent.

Considerable antibacterial efficiency was observed for ZnO, MgO, and Ti_2_O and attributed to the ability of these nanoparticles to: (i) easily bind and damage the bacterial membrane, (ii) penetrate the cell and bind to a specific target, and (iii) generate reactive oxygen species (ROS) on their surfaces that in turn provoke an enhancement of the intracellular oxidative stress. Compared to the organic molecules at a larger scale, metal oxide nanoparticles reveal enhanced temperature stability and may attack bacteria via multiple molecular mechanisms. As a consequence, bacteria are unlikely to develop resistance against them since a series of mutations of the microorganism would be necessary in order to become resistant to the treatment.

Recent advances in nanotechnology of antibacterial nanoparticles include incorporation of metal oxide nanoparticles, especially TiO_2_, CuO, and ZnO into diverse industrial, medical, and household products. For instance, surfaces of dental and other implants coated with TiO_2_ nanoparticles photocatalytically oxidize various bacterial species, preventing postoperative infections (Rasmusson et al. 2005). However, concern has been raised about the biocompatibility of TiO_2_ nanoparticles (Lai et al. 2008). Nowadays, studies are more focused on nanometer-sized metal oxides that are assumed not to be toxic and exhibit antibacterial activity that is not necessarily photoinduced as in the case of TiO_2_. These comprise nanostructured ZnO, MgO, or CaO.

Nano-MgO was shown to exhibit bacteriocidal activity which is highly dependent on the particle size and concentration (Huang et al. 2005; Makhulf et al. 2005) and to act against both Gram-positive and Gram-negative bacteria (Koper et al. 2002; Krishnamoorthy et al. 2012; Makhulf et al. 2005). These nanoparticles are considered as a promising novel antibacterial agent, being harmless to mammalian cells and the environment. For instance, nano-MgO—alone or in combination with other microbials—was proposed as a bacteriocide for treatment of food products in order to improve microbiological food safety (Jin and He 2011).

Nanostructured ZnO is a highly efficient antibacterial agent at significantly low concentrations showing, thus, an advantage compared to nano-MgO. It was also found to act against both, Gram-positive and Gram-negative bacteria (Apperlot et al. 2009; Brayner et al. 2006; Padamavathy and Vijayaraghavan 2008; Stoimenov et al. 2002; Zhang et al. 2007). The mechanism proposed implies that ZnO nanoparticles induce production of ROS, bind to the bacterial membrane and penetrate into bacterial cells. This cytotoxic effect was found to be size-dependent: the smaller the particle size, the greater the efficiency in inhibiting bacterial growth (Apperlot et al. 2009). Despite numerous benefits regarding ZnO antibacterial effects, some recent reports point out that nano-ZnO may exhibit toxic effects on human cells (Lai et al. 2008; Lyon et al. 2007; Taylor and Webster 2011).

We studied the antibacterial effects and toxicity on mammalian cells of ZnMgO nanoparticles. The aim was to combine the strong antibacterial activity of ZnO with safe-to-use antibacterial activities of MgO. We focused on *E. coli* (Gram-negative) and *B. subtilus* (Gram-positive) cultures and compared antibacterial activities of ZnMgO nanoparticles to those of nano-MgO and nano-ZnO. Finally, toxicity on mammalian cells was investigated for all three oxides. Obtained data give the first indication that nano-ZnMgO mixed oxides can be used as antibacterial agents and point to some synergistic effects of its pure metal oxide components.

## Experimental

### Nanoparticles synthesis and characterization

Pure metal oxide nanoparticles (MgO and ZnO) were produced by burning corresponding metal ribbons (Mg and Zn; 99.9 %, Goodfellow) in a glove box made of stainless steel and rigid plastic which is designed to afford vacuum (P ~ 1 mbar). For the production of ZnMgO, a 5 wt% Mg/Zn alloy (Mg_90_/Zn_10_ 5 wt%, Goodfellow) was used. The combustion of metal, i.e., alloy ribbons was started by a thin Ni–Cr wire held in contact with the extremity of the ribbon and which could be resistively heated. For the purpose of subsequent transmission electron microscopy (TEM) measurements, a tweezer-type support holding TEM grid was kept at a constant height (~10 cm) above the sampling point, allowing the collection of particles near the generation zone. The TEM analysis of the particles was achieved by using a JEOL 2100 field emission transmission electron microscope operated at 200 kV with a 0.18 nm resolution. Before any type of measurement, i.e., microscopic investigations and antibacterial and toxicological tests, powders were kept and transported under a vacuum (P ~ 10^−5^ mbar) in order to prevent any contact with the ambient air. X-ray diffraction measurements were performed using a PANalytical X’Pert PRO MRD diffractometer operated at 40 kV and 30 mA by means of the Cu Kα radiation and with the scanning rate of 0.002941°/s. For the analysis of diffraction patterns, we used X’Pert High Score Plus which is a suitable program for powder diffraction and allows research in crystallographic data base. Diffuse reflectance UV spectra were collected on a Varian Cary 5000 UV–Vis–NIR spectrophotometer and Kubelka–Munk model was used to derive the absorbance values from the respective reflectances.

### Bacteria and antibacterial test

*Escherichia coli* strain BL21 DE3 (Invitrogen, France) and *Bacilus subtilis* 168 strain (kindly supplied by D. Dobrijevic; MICALIS, INRA France) were used in this study. Bacteria were cultivated in Luria–Bertani (LB) medium containing 5 g/L of yeast extract, 10 g/L bactotryptone, and 10 g/L NaCl. The saturated cultures were diluted in fresh LB medium to initial optical density (OD600) of 0.1 at 600 nm and incubated in a shaking incubator (200 rpm) at 37 °C. Bacteriological tests were performed by measuring the growth curve of liquid bacterial solutions incubated with nanoparticules. The growth curves were obtained after adding nanoparticle powders to solutions containing *E. coli* or *B. subtilis* (20 mL), by measuring the evolution of optical density as a function of time. OD600 was measured in 100 μL volume using Biophotometer (Eppendorf). A blank containing the equivalent concentration of nanoparticles in LB medium incubated under the same conditions was used as a control.

### Transmission electron microscopy on bacteria

Bacterial cells at OD_600_ = 0.2 were mixed with nanoparticles (1 mg/mL) and incubated at 37 °C for 5 h under shaking (agitation 150 rpm). Ultrastructural visualization of bacterial cells was carried out under a Zeiss 902 electron microscope. Bacterial cells were collected on formal coated copper grids (Agar). After deposition of bacterial suspension on the grids, grids were washed twice with 10 mM sodium acetate buffer, pH 5, and fixed with 2 % glutaraldehyde in PBS (phosphate buffered saline) for 30 s. They were, then, rinsed twice with sodium acetate buffer for 30 s, and finally negatively stained with 1 % uranyl acetate water solution for 30 s. Grids were air-dried before observation.

### HeLa cells and cell death analysis

Human cervical epithelial HeLa cells (ATCC, USA) were cultured in minimum essential medium (MEM) supplemented with Earle’s Salts without l-glutamine (PPA The Cell Culture Company, Austria), completed with 10 % heat-inactivated fetal calf serum (Perbio), 2 mM l-glutamine, penicillin (100 units/mL), and streptomycin (0.1 mg/mL). Cells were grown at 37 °C and 5 % CO_2_.

Cell death in HeLa cells was quantified by acridine orange staining followed by flow cytometry analysis (Becton FACSCalibur, Dickinson and Company, USA) with the 488 nm laser line and FL-1 channel. The cells were trypsinized and centrifuged with the cell culture medium at 3,000×*g* for 5 min. Collected cells were washed two times in PBS, then, resuspended in MEM containing acridine orange (0.1 μg/mL) and incubated at 37 °C for 10 min in the dark. Stained cells were collected and washed two times with PBS, and, then, fixed with 3.2 % PFA (paraformaldehyde) in PBS for 30 min. For analysis, the fixed cells were collected at 5,000×*g* for 6 min, and resuspended in PBS. The analysis was done on 5 × 10^4^ cells.

The putative changes in the morphology of HeLa cells treated with nanoparticles were compared to that of corresponding untreated cells by acquiring bright field images using Nikon TE200 inverted microscope equipped with a Photometrics CoolSNAP ES2 camera. Images were processed using MetaVue software (Molecular Devices).

## Results and discussion

### Synthesis and characterization of metal oxide nanoparticles

**Fig. 1:**
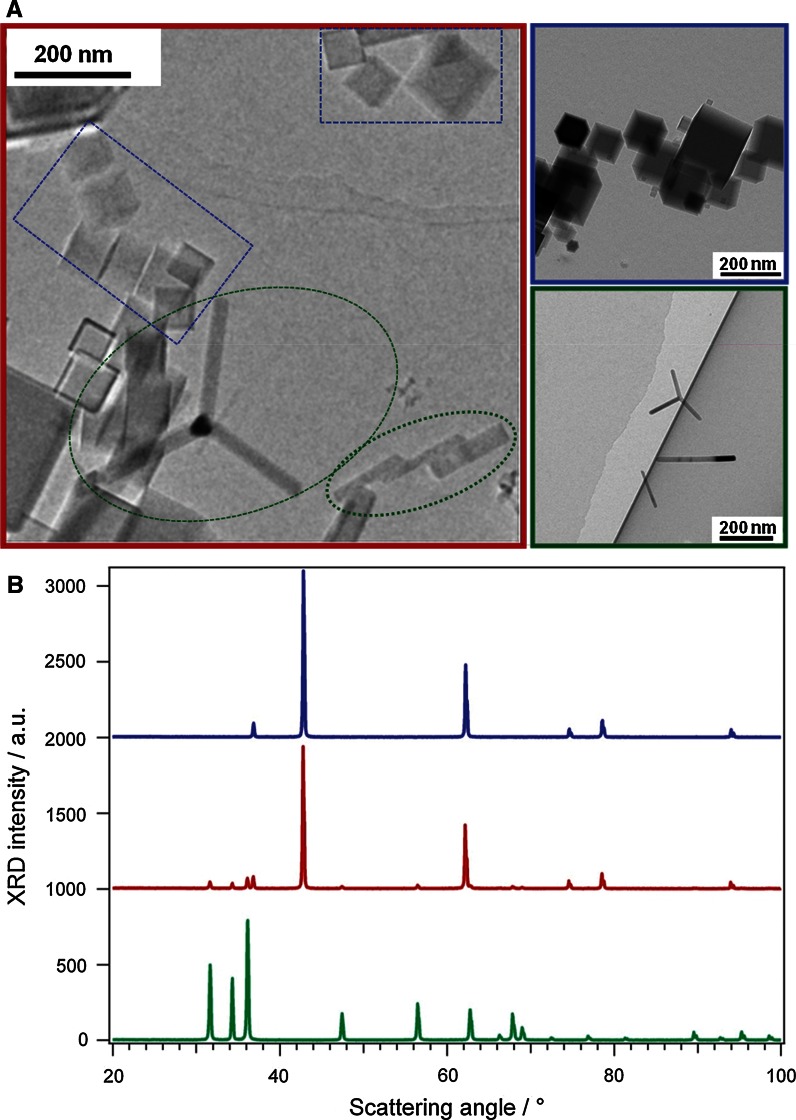
TEM images (**a**) and X-ray diffraction patterns (**b**) of ZnO (*green*), MgO (*blue*), and ZnMgO (*red*) nanoparticles

The morphology of nanoparticles was characterized by means of transmission electron microscopy. Representative images of all three oxides are shown in Fig. 1a. MgO nanoparticles (image with blue outline) adopt regular cubic shape with an average particle size of ~50 nm that is typical for the synthesis condition applied here (Stankic et al. 2011). Combustion of metallic Zn in the air atmosphere results in nanoparticles with two different shapes: nanorods and tetrapods (image with green outline). In tetrapods, four arms project out at tetrahedral angles from a branching point but the fourth “arm” is not visible on the selected photo. Description is, however, based on several TEM images taken with different angles (not shown here). Length of either nanorods or tetrapod arms varies between 150 and 200 nm, whereas the corresponding diameters were measured to be ~10 nm. TEM image recorded on ZnMgO nanopowder (red outlined) shows that in addition to cubically shaped nanoparticles (marked with blue rectangles) also tetrapods and nanorods (marked with green) were found. This implies that the ZnMgO powder consists of structures which are characteristic for its pure components—MgO and ZnO. XRD patterns acquired on ZnO (green) and MgO nanopowders (blue) fit perfectly to hexagonal and cubic structure, respectively (Fig. 1b) while in the ZnMgO diffractogram (Fig. 1b, red) both, ZnO- and MgO-specific reflexes were present. The similarity in ionic radii between Mg^2+^ (1.36 Å) and Zn^2+^ (1.25 Å) allows significant replacements in either structure. Although the ZnMgO phase diagram (Segnit and Holland 1965) and the wt% of ZnO in our mixed sample (less than 5 %) correspond to cubic crystal structure of the mixed oxide both, TEM images and XRD unambiguously demonstrate that the phase separation—most probably as a consequence of synthesis technique applied here—occurs in the mixed oxide.

**Fig. 2:**
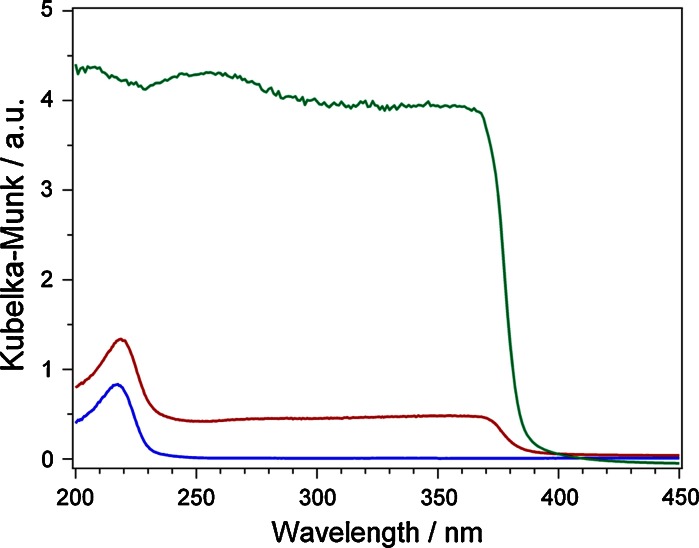
Room temperature diffuse reflectance UV/Vis spectra of ZnO (*green*), MgO (*blue*), and ZnMgO (*red*) nanoparticles

UV/Vis diffuse reflectance (optical absorption) spectra of MgO, ZnO, and composite ZnMgO nanopowders are presented in Fig. 2. In comparison to the UV diffuse reflectance spectra of the MgO sample (blue curve), the spectrum of the ZnMgO sample shows Zn^2+^-induced changes in the absorption properties (red curve). These changes are either characterized by a slightly red-shifted absorption band or an absorption threshold at 3.4 eV which is consistent with the band gap of ZnO (green curve) (Klingshirn 2007). The latter observation is supported by previously discussed XRD and TEM results and conclusion that the phase separation into MgO-rich cubic phase and ZnO-rich wurtzite phase should have occurred during the combustion synthesis where the particles are exposed to high temperatures. Although two phases are observed in TEM images (Figs. 1, 2) we cannot totally rule out the possibility that Zn is partially integrated into MgO cubes and vice versa. However, regarding the relatively week percentage of Zn (less than 5 %) and the appearance of particle shapes typically found in pure powders—tetrapods in ZnO and cubes in MgO—we assume that ZnMgO powder mainly consists of pure and separated components. Finally, also energy-dispersive X-ray spectroscopy has shown no presence of Zn-atoms in MgO cubes and vice versa but, since this method is typically sensitive down to levels of 0.1 atomic percent we cannot exclude the existence of solid solutions up to this percentage range.

### Antibacterial effects of ZnO, MgO, and ZnMgO nanoparticles

First, we tested the antimicrobial activities of nanoparticles at different concentrations (0.001—1 mg/mL) against *E. coli* and *B. subtilis* in LB medium (data not shown). After 5 h treatment, nano-ZnO-inhibited growth of both bacteria in a concentration-dependent manner, showing complete bacteriocidal effect for concentrations above 0.1 mg/mL. In contrast, bacterial incubation for 5 h with nano-MgO or nano-ZnMgO resulted in a relatively small rate of bacterial growth inactivation for a concentration lower than 1 mg/mL. Thus, to further compare antibacterial effects of nanoparticles, concentrations of 1 mg/mL were used for all three types of metal oxide nanoparticles.

**Fig. 3:**
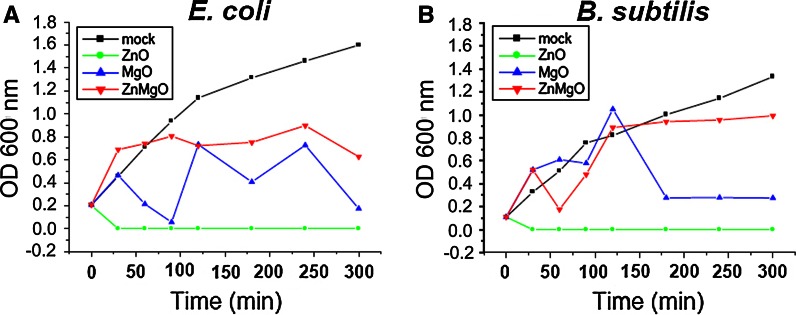
Growth curves of *E. coli* (**a**) and *B. subtilis* (**b**) in Luria–Bertani (LB) medium in the presence of 1 mg/mL: ZnO (*green*), MgO (*blue*), or ZnMgO (*red*) nanoparticles

Figure 3 shows kinetic effects of the nano-metal oxides on *E. coli* and *B. subtilis* growth curves. Within less than 1 h, ZnO nanoparticles completely eradicated bacteria in both cultures, whereas MgO nanocubes reduced viability of *E. coli* and *B. subtilis* by 89 and 78 %, respectively, after 5 h of treatment. Fluctuations of optical density in Fig. 3 originated from bacterial precipitation and variation of the cell number charged into the spectroscopic cuvette. The smallest antibacterial efficiency was found for ZnMgO nanoparticles; the viability of *E. coli* and *B. subtilis* was found to be reduced by 61 and 25 %, respectively, after 5 h treatment. This result suggests that the mixed oxide exhibits reduced kinetics of antibacterial activity with respect to either pure component.

**Fig. 4:**
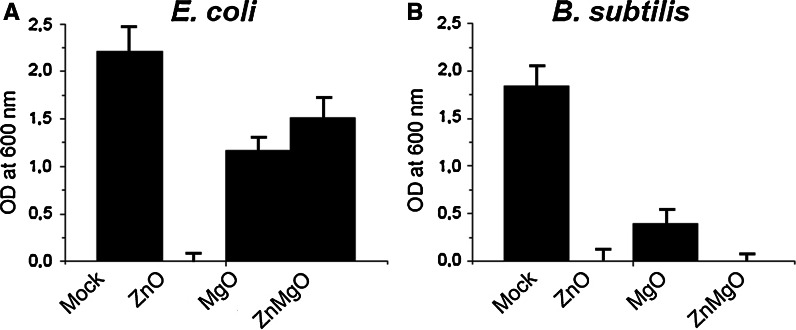
Antibacterial efficiency of ZnO, MgO, and ZnMgO nanoparticles at 1 mg/mL tested on *E. coli* (**a**) and *B. subtilis* (**b**) after 24 h incubation. Note that ZnO completely eliminates both bacteria’s strains while ZnMgO eliminates only *B. subtilis*

We then quantified bacterial viability after 24 h incubation with nanoparticles (Fig. 4). As expected, there were no surviving bacteria in the medium containing ZnO nanoparticles. Treatment with nano-MgO particles resulted in 53 % survival of *E. coli*, and only 21 % of *B. subtilis*. Surprisingly, under the same conditions ZnMgO nanoparticles completely inactivated the growth of *B. subtilis*, while about 80 % *E. coli* survived this treatment. Comparing these to the results obtained in kinetic tests above, it is obvious that the ZnMgO system exhibits different antibacterial kinetic with respect to pure ZnO and needs more than 5 h to be sufficiently active. Moreover, this result indicates not only the high antibacterial activity of ZnMgO mixed system but it implies also its selectivity against Gram-positive bacteria. This may be explained by differences in cell wall structure between Gram-positive and Gram-negative bacteria implying that ZnMgO exhibits higher binding affinity to Gram-positive bacteria cell wall. Several mechanisms have been reported for the antibacterial activity of metal oxide nanoparticles. One of these is to bind to the cell membrane of microorganisms due to their high affinity to interact with membrane lipids, such as phospholipids (Krishnamoorthy et al. 2012; Zhang et al. 2007). When interacting with the membrane, nanoparticles can disorganize the membrane structure and dynamics. This leads to the lost of membrane integrity, malfunction, and finally to bacterial death. Being Gram-positive bacteria *B. subtilis* has only a single unit lipid membrane, in contrast to Gram-negative *E. coli* which has a cytoplasmic membrane and an outer cell membrane. We may, therefore, suggest that selective antibacterial activity of nano-ZnMgO against *B. subtilis* is, when compared to *E. coli*, related to specific bacterial membrane lipid composition and total charge, and/or different membrane layer thickness and structure.

There are, furthermore, studies which have shown that at nanoscale level ZnO and MgO may also penetrate into bacterial cells and produce there toxic oxygen radicals (Apperlot et al. 2009; Irzh et al. 2010; Makhulf et al. 2005; Zhang et al. 2007). In aqueous solutions, both MgO and ZnO nano-components produce peroxides that may act as oxidizing agents. These ROS can damage DNA, cell membrane, or cell proteins, and may lead to the inhibition of bacterial growth and eventually to bacterial death (Moody and Hassan 1982). Some bacteria are more resistant to oxidative stress by producing protective enzymes, such as super oxide dismutase or catalase which neutralize reactive oxidative species. To elucidate between the two proposed mechanisms, interaction of water with ZnMgO needs to be studied. This is, however, in the domain of surface science chemistry and exceeds the scope of the present paper.

**Fig. 5:**
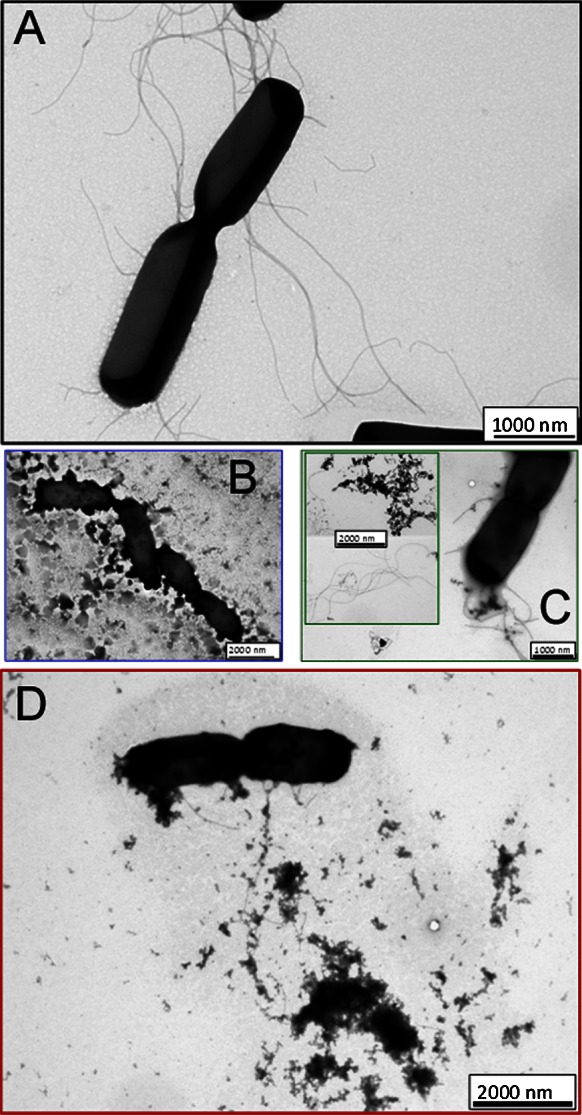
TEM images of untreated *B. subtilis* (**a**) and treated *B. subtilis* with ZnO (**b**), MgO (**c**), and ZnMgO (**d**) nanoparticules (for 5 h, at concentration 1 mg/mL)

Transmission electron microscopy was applied in order to evidence possible changes at bacterial membranes upon their interactions with metal oxide nanoparticles. In medium free of metal oxide nanoparticles, *B. subtilis* were in linear phase of multiplication, so that the most bacteria observed were in division, as illustrated in Fig. 5a. The untreated bacterial cells were in rod shapes of normal size of about 1 μm with intact cell structure. In Fig. 5b–d, we show TEM images of *B. subtilis* treated for 5 h with ZnO, MgO, and ZnMgO nanocrystals. Admixture of MgO nanocubes to the medium-induced morphological changes of bacteria cells at the membrane level (Fig. 5b). Those changes reflect the formation of irregular cell surface and alteration of cell membrane. However, *B. subtilis* were observed in cell division phase, as untreated bacteria, indicating that crucial steps in cell division process were not inhibited by the presence of MgO nanocubes.

Curiously, when ZnO nanocrystals were added to the bacterial solution, almost no integral cell was observed. Instead, flagella disconnected from bacteria can be seen as well as some high density material, suggesting the leakage of the internal cell contents (Fig. 5c). A similar effect of ZnO nanoparticles on bacterial cell walls was previously shown by Brayner et al. (2006). We hypothesize that significantly sharp shapes of our ZnO nanorods and nanotetrapods, both found to have ~10 nm diameter, easily damage the membrane integrity allowing in addition faster penetration of nanoparticles into bacterial cells. Moreover, TEM images insert in Fig. 5c show flagella associated with nanoparticles suggesting that this metal oxide binds not only membrane lipids but also proteins from flagella.

Finally, TEM analysis showed that ZnMgO nanoparticles cause extensive injury of the bacterial cell membrane and complex morphological changes. As illustrated in Fig. 5d, bacterial cells treated with nano-ZnMgO shrunk to smaller and more rounded shapes than untreated cells (Fig. 5a). In addition, the cell membrane appears to have a more irregular rough surface. The inhomogeneous appearance of the cytoplasm might be a result of the leakage of the cell content. Moreover, some cellular debris associated with the nanoparticles was also detected. This finding also implies that nano-ZnMgO possibly provokes an internal cellular content leak. Complementary studies are needed to elucidate the molecular mechanism by which mixed ZnMgO damages bacteria.

## Cytotoxicity analysis

**Fig. 6:**
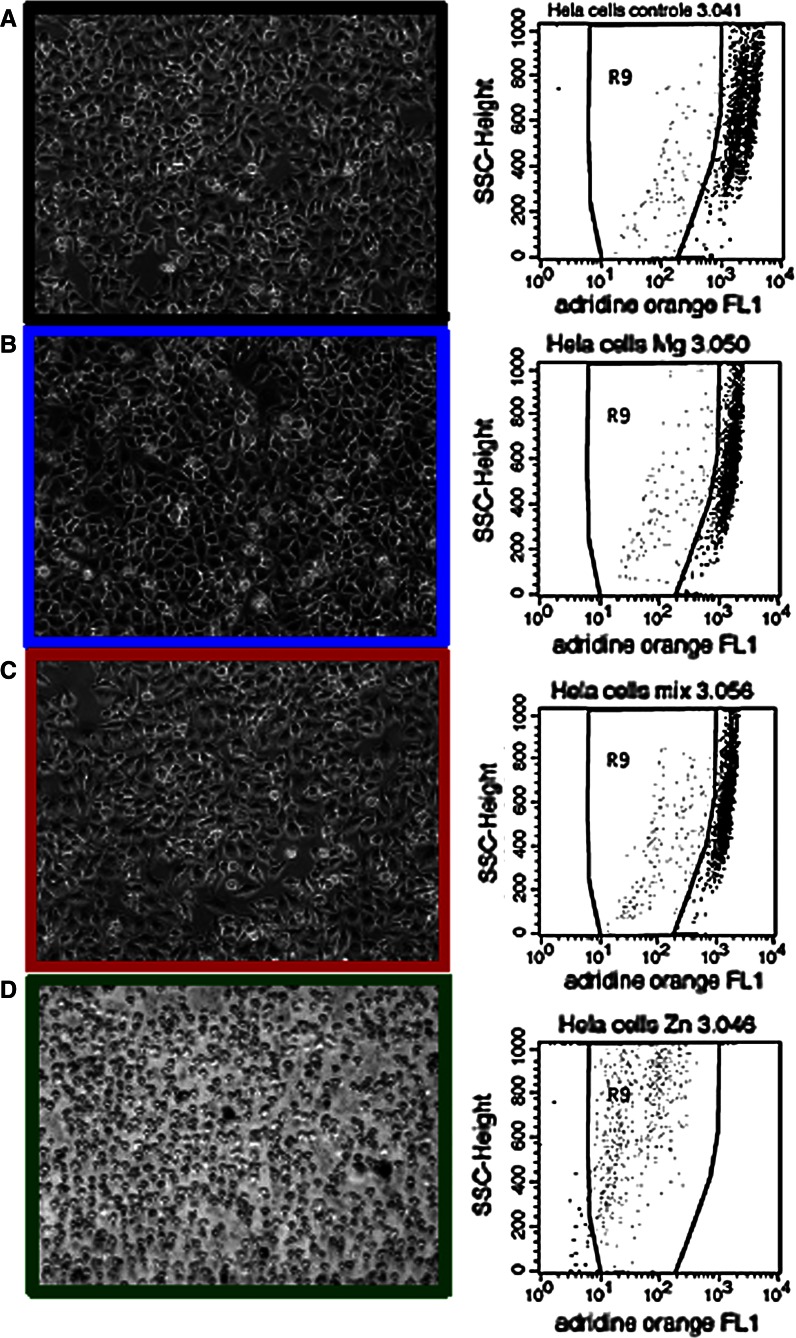
*Left panel* optical images of (**a**) untreated HeLa cells (Mock) and HeLa cells treated with (**b**) MgO (*blue outline*), (**c**) ZnO (*green outline*), and (**d**) ZnMgO nanoparticles (24 h, 1 mg/mL). *Right panel* quantification of the cytotoxic effect of nanoparticles on HeLa cells by acridine orange staining and flow cytometry analysis

Nanoparticles are promising agents for antibacterial applications only if they are toxic to bacteria but not mammalian cells. Indeed, some antibacterial nanoparticles can be phagocytosed and subsequently degraded by lysosomal fusion and thus appear non-toxic to mammalian cells (Arbab et al. 2005; Taylor and Webster 2011). We used HeLa cells to probe the cytotoxic effects of ZnO, MgO, and ZnMgO on mammalian cells. The cells were treated with metal oxide nanoparticles at a concentration of 1 mg/mL for 24 h, at optimal conditions for antibacterial activity. Treated HeLa cells were firstly analyzed via optical microscopy. Figure 6 left panel displays representative images of treated cells. No changes with respect to the morphology and density of HeLa cells could be observed after incubations with nano-MgO or nano-ZnMgO, whereas the ZnO treatment led to the death of all cells. To support these findings, a quantification of cell death was performed on treated HeLa cells (24 h incubation) by acridine orange staining (Darzynkiewicz and Kapuscinski 1990) and flow cytometry analysis (Fig. 6 right panel). The reduction of acridine orange derived fluorescence intensity in cells indicated that ZnO nanoparticles induce cell death in more than 97 % of treated HeLa cells. On the contrary, only about 10 % of cell mortality was observed after treatment with either nano-MgO or nano-ZnMgO. In control experiments, untreated HeLa cells presented about 5 % cell mortality. The quantitative cell death analysis data are, therefore, consistent with the observation made by optical microscopy, suggesting that MgO and ZnMgO nanoparticles—when compared to ZnO nanoparticles—are significantly less toxic to mammalian cells. These results are perfectly in line with the previous study on pure nano-ZnO and nano-MgO cytotoxic effect on human neural cells and fibroblasts (Lai et al. 2008). They found that nano-ZnO was more effective in inducing cellular death than nano-MgO.

Overall, our results suggest that ZnMgO nanoparticles possess a strong antibacterial effect toward Gram-positive bacteria but do not affect the cell viability of HeLa. TEM analysis implies that ZnMgO nanocrystals bind to bacterial membrane causing membrane disruption. On the contrary, HeLa cells found the way to neutralize their toxic effect and there is no membrane damage. We assume that the HeLa cell uptakes ZnMgO nanoparticles and metabolize them—which finally leads to the neutralization of their toxicity. Similar effect was shown for iron-oxide nanoparticles which can be internalized into enosome/liposomes and degraded there (Arbab et al. 2005). Nano-Ag_2_O_3_ is, also, not toxic to mammalian fibroblast L929 and BJ cells at concentrations having antibacterial activity (Radziun et al. 2011). Nano-Ag_2_O_3_ of 50–80 nm size penetrates fibroblast cells without affecting cell viability or inducing apoptosis. Similarly, when nano-Ag_2_O_3_ was used to stabilize nano-Ag or nano-Pr, no phyto- or eco-toxicity was observed (Jastrzębska et al. 2012). Our cytotoxic analysis suggests no toxic effect of ZnMgO, and thus potentially its good biocompatibility. Further detailed study is, however, needed to elucidate both, the antibacterial mechanism and significant selectivity of ZnMgO nanoparticles.

## Conclusions and perspectives

Antibacterial and toxicological impacts of ZnMgO mixed nanoparticles were studied and compared to those of pure ZnO and MgO nanoparticles. For this purpose, pure nanoparticles as well as their mixture—with Zn content lower than 5 wt%—were synthesized via a metal combustion technique. Transmission electron microscopy shows that ZnMgO powder consists of nanocubes, nanorods, and nanotetrapods; structures which were also observed in its pure components. Focusing on both, Gram-positive and Gram-negative bacteria, we show that among three studied oxides, ZnO nanorods and nanotetrapods reveal the highest antibacterial efficiency while being toxic to mammalian cell—as demonstrated on HeLa cells. On the contrary, MgO nanocubes inhibit only partially bacterial growth being at the same time harmless to mammalian cells. Finally, mixed ZnMgO nanoparticles reveal synergetic effects of the pure components: no damages on mammalian cells were observed—similarly as for pure MgO whereas they show strong antibacterial activity—comparable to pure ZnO—which is in addition highly selective for Gram-positive bacteria. Complementary studies are in progress and they aim to: (i) monitor bacterial growth in the presence of ZnMgO varying Zn/Mg ratio, sizes and shapes, (ii) determine the molecular mechanism of antibacterial effects of nano-ZnMgO, and (iv) extend toxicological studies on other types of mammalian cells.
